# Assessment of the Relationship Between Liver Enzymes and Cardiovascular Disease: A Study in Bangladeshi Adults

**DOI:** 10.1002/edm2.481

**Published:** 2024-03-17

**Authors:** Akibul Hasan, Ali Newaj, Aporajita Das Trisha, Jaasia Momtahena Hafsa, Nayan Chandra Mohanto, Nurshad Ali

**Affiliations:** ^1^ Department of Biochemistry and Molecular Biology Shahjalal University of Science and Technology Sylhet Bangladesh

**Keywords:** association, Bangladesh, CVD, liver enzymes

## Abstract

**Objectives:**

Elevated liver enzyme levels are suggested to be associated with an increased risk of cardiovascular disease (CVD). However, few studies have explored the relationship between liver enzymes and myocardial infarction (MI). This study aimed to evaluate the potential association of elevated liver enzymes with MI within a population group in Bangladesh.

**Methods:**

In this cross‐sectional study, 348 participants were enrolled, 189 with MI in the CVD group and 159 in the control group. Serum levels of liver enzymes (AST, ALT and GGT) and other biochemical parameters were measured using standard methods. Multivariate logistic regression models were applied to determine the associations between elevated liver enzymes and CVD.

**Result:**

In the CVD group, 51.6%, 30.9% and 67.7% of individuals had elevated serum AST, ALT and GGT levels, respectively. On the contrary, the control group had 17.0%, 15.1% and 35.2% of individuals with high serum AST, ALT and GGT levels, respectively. Overall, 71.8% of the subjects in the CVD group and 44.7% of the subjects in the control group had at least one or more elevated liver enzymes (*p* < 0.001). The mean level of all three liver enzymes was significantly higher in the CVD group than in the control group (*p* < 0.001). In both the CVD and control groups, males had higher levels of liver enzymes than females. In the regression models, the serum levels of AST, ALT and GGT showed a positive and independent association with the prevalence of CVD (*p* < 0.001). However, GGT showed the strongest association among the three enzymes.

**Conclusions:**

This study shows a high prevalence of liver enzyme abnormalities in individuals with CVD. Serum levels of AST, ALT and GGT were independently associated with the prevalence of CVD. This suggests that measuring liver enzyme levels could be a useful marker in predicting CVD at an early stage.

## Graphical abstract

This study shows a high prevalence of liver enzyme abnormalities in CVD patients. Serum levels of AST, ALT and GGT were independently associated with the prevalence of CVD.

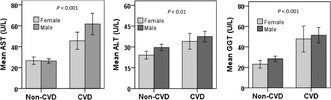

## 1 Introduction

Liver enzymes such as aspartate aminotransferase (AST), alanine aminotransferase (ALT) and γ‐glutamyltransferase (GGT) are widely used as markers of hepatic damage and nonalcoholic fatty liver disease (NAFLD) [1]. NAFLD is closely related to obesity, insulin resistance, diabetes and metabolic syndrome [1]. Over the last few decades, these liver enzymes have drawn significant attention as an emerging marker of cardiovascular disease (CVD) risk; however, uncertainty exists because the questions regarding their aetiological role in CVD remain unclear. Whereas several studies addressed a relationship between liver enzymes and CVD risk [2, 3, 4, 5, 6], others have shown minimal effects or no relationship, and results are inconsistent across studies [7, 8, 9, 10, 11]. A meta‐analysis by Fraser et al. [8] demonstrated that GGT but not ALT is related to coronary heart disease (CHD) and stroke. Another stratified analysis of prospective cohort studies has shown that ALT is negatively associated with CHD but positively related to stroke [12]. On the contrary, Lee et al. [4] showed a positive association of AST and ALT with CVD and all‐cause mortality. Further studies showed a positive relationship between liver enzymes (ALT, AST and GGT) and CVD and all‐cause mortality [3]. The variation in these study findings might be related to differences in sample size, age, sex, ethnicity and variability in disease conditions [5]. Nevertheless, exploring the relationship between liver function test markers and CVD risk factors is worthwhile, as early CVD prevention and management may significantly impact individuals and society.

Bangladesh is a developing country in South Asia. The prevalence of noncommunicable chronic diseases and associated mortality has significantly increased in this country over the past decades because of fast economic growth, rapid urbanisation, industrialisation and increased consumption of unhealthy diets [13, 14, 15]. In Bangladesh, CVD accounts for 30% of deaths, whereas noncommunicable diseases are estimated to account for 67% of all deaths [16]. Although liver dysfunction is associated with an increased risk of CVD, there is no information about the relationship between elevated liver enzymes and CVD in the Bangladeshi population. This study aimed to investigate the relationship between elevated levels of liver enzymes and the prevalence of myocardial infarction (MI), a major type of CVD in Bangladeshi adults.

## 2 Methods

### 2.1 Study Area and Participants

For this study, blood samples and data were collected between February 2019 and January 2020. The participants with MI in the CVD group (*n* = 189) were recruited from Osmani Medical College Hospital, Sylhet, and participants without CVD in the control group (*n* = 159) were recruited from the general population of the Sylhet region in Bangladesh. The participant with MI was identified using the International Classification of Diseases, 10th revision (ICD‐10) codes I21‐I22, which were also confirmed by baseline electrocardiogram (ECG) or medical records obtained during the baseline interview. As inclusion criteria, the study included both males and females who were over 18 years old and had suffered from a MI. Pregnant and nursing women, as well as individuals who had previously been diagnosed with hepatic, renal or infectious diseases, were excluded. All participants provided written informed consent before taking part in the study. The study protocol (reference no. 02/BMB/2019) was approved by the Ethics Committee at the Department of Biochemistry and Molecular Biology, School of Life Science, Shahjalal University of Science and Technology. The study was conducted in compliance with relevant guidelines and regulations.

### 2.2 Anthropometric and Blood Pressure Data

Anthropometric data including weight, height and blood pressure were measured following the standardised protocols described elsewhere [17, 18, 19, 20]. Weight was measured in light clothing without shoes using a digital scale (Beurer 700, Germany), and standing height was measured without shoes using a tape meter. Body mass index (BMI) was calculated as weight divided by height in metres squared (kg/m^2^). Systolic and diastolic blood pressure (SBP and DBP, respectively) were measured twice after 10 min of rest in the seated position and averaged using a digital sphygmomanometer (Omron M10; Omron Corporation, Tokyo, Japan). The participants ‘physical activity and smoking status’ were also included in the questionnaire form.

### 2.3 Laboratory Analysis

Approximately 5 mL of blood samples was collected from the participants with the support of expert personnel. After collection, the blood samples were quickly transported to the clinical biochemistry laboratory. Serum samples were separated after centrifugation and stored at −20°C until targeted markers analysis. Colorimetric enzymatic assays were used to measure the serum concentrations of liver enzymes (ALT, AST and GGT), lipid profile (TC, TG, LDL and HDL), glucose and creatinine. Diagnostic kits were purchased from Human Diagnostic (Germany) except GGT (Vitro Scient, Egypt). All the analyses were performed with a Biochemistry Analyser (Humalyzer 3000; USA). The accuracy of the method was maintained using the reference standards.

### 2.4 Diagnostic Criteria

The participant with MI was defined according to the International Classification of Diseases, 10th revision (ICD‐10) codes (I21‐I22) and confirmed by baseline ECG or medical records in baseline interview. The abnormal liver enzyme levels were defined as one or more measurements of AST > 35 U/L in men/> 31 U/L in women, ALT > 45 U/L in men/> 34 U/L in women and GGT > 55 U/L in men/> 38 U/L in women [21].

### 2.5 Statistical Data Analysis

Numerical variables are expressed as mean ± SD, and nominal variables are presented as frequencies (%). The prevalence of elevated liver enzymes in the sex groups was determined by a chi‐squared test. The differences in variables mean between the groups were analysed by independent sample *t*‐test. The association between elevated liver enzymes and CVD was evaluated by applying three models in the multivariate logistic regression analysis. All statistical results were analysed using IBM SPSS version 23, and *p*‐values below 0.05 are considered statistically significant.

## 3 Results

### 3.1 Characteristics of the Study Participants

**FIGURE 1:**
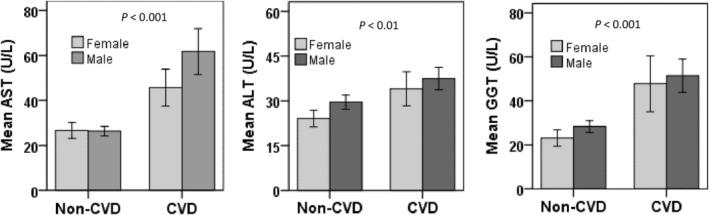
Level of liver enzymes in the non‐CVD and CVD groups according to gender. *p*‐Values are obtained from independent sample *t*‐test. ALT, alanine aminotransferase; AST, aspartate aminotransferase; CVD, cardiovascular disease; GGT, γ‐glutamyltransferase.

**FIGURE 2:**
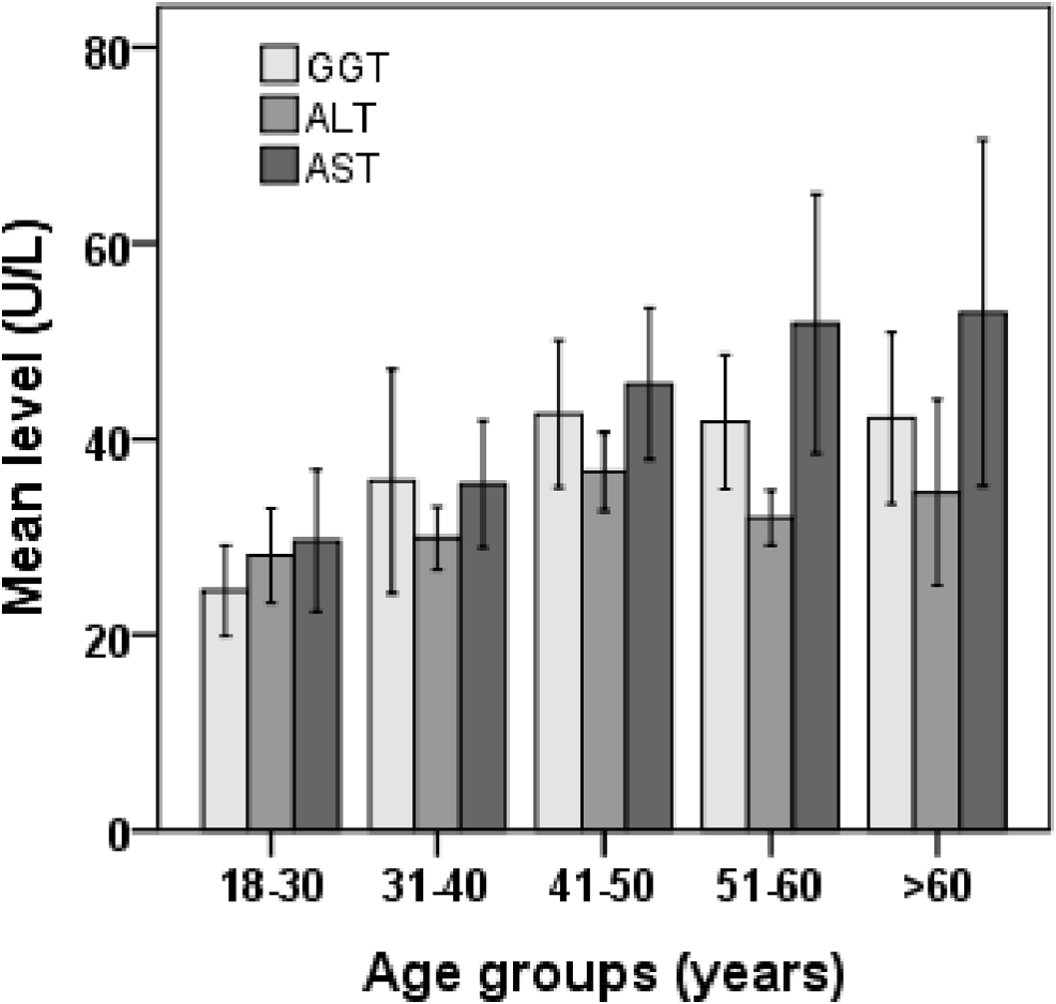
Level of liver enzymes in different age groups. ALT, alanine aminotransferase; AST, aspartate aminotransferase; GGT, γ‐glutamyltransferase.

The characteristics of the study participants are presented in Table 1. Among the participants, 74.1% were male and 25.9% were female. The average age of the participants in the CVD group was 52.3 ± 11.2 years, while in the control group, it was 43.9 ± 15.1 years (*p* < 0.001). The average BMI level was lower in the CVD group (21.4 ± 3.1 kg/m^2^) than in the control group (24.1 ± 3.8 kg/m^2^) (*p* < 0.001). The mean concentrations of liver enzymes were significantly higher in the CVD group (AST 58.1 ± 56.8 U/L, ALT 36.7 ± 22.1 U/L and GGT 50.3 ± 40.6 U/L) than in the control group (AST 26.5 ± 11.3 U/L; ALT 27.9 ± 11.7 U/L; and GGT 26.7 ± 13.6 U/L) (*p* < 0.001). Male participants in both the CVD and control groups had higher levels of liver enzymes than females (Figure 1). When the participants were divided into different age groups, it was found that liver enzymes, especially AST, increased steadily with age (Figure 2). An increase in liver enzyme levels was observed in participants over 40 years old compared with those under 40 years old. A significant difference was observed in smoking status and physical activity between the CVD and control groups.

**TABLE 1:** Characteristics of the participants in the CVD and non‐CVD (control) groups.

Variable	Control (non‐CVD)	CVD	*p*‐Value
Number (*N*)	159	189	—
Gender, *n* (%)			
Male	109 (68.6)	149 (78.8)	—
Female	50 (31.4)	40 (21.16)	—
Age	43.9 ± 15.1	52.3 ± 11.2	<0.001
BMI (kg/m^2^)	24.1 ± 3.8	21.4 ± 3.1	<0.001
SBP	130.9 ± 18.3	117.1 ± 23.9	<0.001
DBP	81.3 ± 9.8	75.8 ± 14.1	<0.001
Glucose (mmol/L)	7.7 ± 4.31	5.9 ± 2.5	<0.001
AST (U/L)	26.5 ± 11.3	58.1 ± 56.8	<0.001
ALT (U/L)	27.9 ± 11.7	36.7 ± 22.1	<0.001
GGT (U/L)	26.7 ± 13.6	50.3 ± 40.6	<0.001
Creatinine (mg/dL)	0.8 ± 0.4	0.9 ± 0.7	0.264
TG (mg/dL)	195 ± 124.4	139 ± 77.8	<0.001
TC (mg/dL)	227 ± 92.5	144 ± 41.4	<0.001
HDL (mg/dL)	34.9 ± 15.5	20.2 ± 11.7	<0.001
LDL (mg/dL)	153 ± 85.4	95 ± 41.8	<0.001
Smoking status, *n* (%)			
Yes	44 (27.7)	128 (67.7)	<0.001
No	115 (72.3)	61 (32.3)	
Physical activity, *n* (%)			
Low	33 (20.7)	134 (70.9)	<0.001
Medium	109 (68.6)	48 (25.4)	
High	17 (10.7)	7 (3.7)	

*Note*: Data are presented as mean ± SD. *p*‐Values are obtained from independent sample *t*‐test when compared between the case and control groups.

Abbreviations: ALT, alanine aminotransferase; AST, aspartate aminotransferase; BMI, body mass index; CVD, cardiovascular disease; DBP, diastolic blood pressure; GGT, γ‐glutamyltransferase; HDL, high‐density lipoprotein cholesterol; LDL, low‐density lipoprotein cholesterol; SBP, systolic blood pressure; TC, total cholesterol; TG, triglyceride.

### 3.2 Prevalence of Elevated Liver Enzyme in the Control and CVD Groups

Overall, 44.7% of the subjects in the control group and 71.8% of the subjects in the CVD group had at least one or more elevated liver enzymes (*p* < 0.001) (Table 2). The frequency of elevated liver enzymes was higher in males than in females in both groups, except for ALT in the CVD group. Among the three liver enzymes, serum GGT showed the highest prevalence of abnormalities in both the control (41.3%) and CVD groups (67.7%) (*p* < 0.001).

**TABLE 2:** Prevalence of elevated liver enzymes in the non‐CVD and CVD groups by gender.

Parameter	Non‐CVD, *n* (%)				CVD, *n* (%)			
	Overall	Male	Female	*p*‐Value	Overall	Male	Female	*p*‐Value
AST	27 (17)	18 (16.5)	9 (18)	0.817	97 (51.6)^a^	80 (54.1)	17 (42.5)	0.195
ALT	24 (15.1)	20 (18.3)	4 (8)	0.091	58 (30.9)^a^	48 (32.4)	10 (25.0)	0.367
GGT	56 (35.2)	45 (41.3)	11(22)	0.018	107 (67.7)^a^	84 (71.2)	23 (57.5)	0.110
At least one enzyme	71 (44.7)	56 (51.4)	15 (30)	0.012	135 (71.8)^a^	107 (72.3)	28 (70)	0.774

*Note*: Data are expressed as *n* (%). In other cases, *p*‐values are determined from the gender differences. *p*‐Values are obtained from chi‐squared test when the prevalence is compared between the sex groups.

Abbreviations: ALT, alanine aminotransferase; AST, aspartate aminotransferase; CVD, cardiovascular disease; GGT, γ‐glutamyltransferase.

^a^ *p* < 0.001, when the prevalence of elevated liver enzymes in the overall group is compared between non‐CVD and CVD.

### 3.3 Regression Analysis to Evaluate the Association Between Liver Enzymes and CVD

In multivariate logistic regression analysis, three models were applied to evaluate the relationship between liver enzymes and MI (Table 3). Model 1 adjusted for age, Model 2 adjusted for parameters in Model 1 as well as BMI, blood pressure and glucose, and Model 3 adjusted for parameters in Model 2, as well as lipid profile, smoking status and physical activity. Overall, in all three models, liver enzymes showed a positive and independent association with the prevalence of MI (*p* < 0.01, at least for all cases). This independent association between liver enzymes and MI was more robust in males than in females. Of the three liver enzymes, serum GGT showed the strongest association with MI.

**TABLE 3:** Association of liver enzymes with the prevalence of CVD.

	AST		ALT		GGT	
	OR (95% CI)	*p*‐Value	OR (95% CI)	*p*‐Value	OR (95% CI)	*p*‐Value
Male						
Model 1	1.06 (1.04–1.08)	<0.001	1.04 (1.02–1.06)	0.000	1.04 (1.03–1.06)	<0.001
Model 2	1.07 (1.04–1.09)	<0.001	1.04 (1.01–1.06)	0.002	1.05 (1.03–1.07)	<0.001
Model 3	1.08 (1.04–1.12)	<0.001	1.04 (1.00–1.07)	0.039	1.05 (1.02–1.08)	0.001
Female						
Model 1	1.05 (1.01–1.09)	0.015	1.04 (1.01–1.08)	0.009	1.04 (1.01–1.07)	0.009
Model 2	1.08 (0.99–1.16)	0.054	1.05 (0.98–1.13)	0.171	1.05 (1.00–1.11)	0.010
Model 3	1.03 (0.94–1.13)	0.510	0.99 (0.90–1.09)	0.879	1.05 (1.00–1.13)	0.010
Overall						
Model 1	1.06 (1.04–1.08)	<0.001	1.04 (1.02–1.06)	0.000	1.04 (1.03–1.06)	<0.001
Model 2	1.07 (1.04–1.09)	<0.001	1.04 (1.02–1.06)	0.000	1.06 (1.04–1.08)	<0.001
Model 3	1.07 (1.04–1.11)	<0.001	1.04 (1.02–1.07)	0.001	1.05 (1.03–1.08)	<0.001

*Note*: In multivariate logistic regression models, the dependent variable was CVD (yes), and the independent variables were AST, ALT and GGT. The reference category is control (non‐CVD). Model 1: adjusted for age and sex. Model 2: Model 1 + BMI, SBP, DBP and glucose. Model 3: Model 2 + lipid profile, smoking and physical activity.

Abbreviations: ALT, alanine aminotransferase; AST, aspartate aminotransferase; BMI, body mass index; CVD, cardiovascular disease; DBP, diastolic blood pressure; GGT, γ‐glutamyltransferase; SBP, systolic blood pressure.

## 4 Discussion

This study explored the relationship between serum liver enzymes and CVD in a population group in Bangladesh. It is worth noting that this is the first study to investigate the association between elevated liver enzymes and CVD in Bangladeshi adults.

In the present study, the prevalence of elevated liver enzymes was significantly higher among subjects with CVD than in subjects without CVD. In regression models, all three liver enzymes showed a positive and independent association with the risk of CVD. Our findings on the association between liver enzymes and CVD are supported by some previous studies. A recent large‐scale population‐based study in Korea indicated that high variability in AST, ALT and GGT was associated with an increased risk for all‐cause mortality and cardiovascular events, and the degree of association was most significant for GGT variability [2]. Another large‐scale study showed a significant relationship between liver enzymes (ALT, AST and GGT) and CVD development and all‐cause mortality in the population of Korea [3], where GGT showed the strongest association among the three liver enzymes [3]. Further observational studies in the same country showed that individuals with elevated AST or ALT levels had increased risks of CVD‐related mortality and all‐cause mortality than those with normal levels of AST or ALT, after adjusting for several confounding variables [4].

Another recent study found significant linear relationships between blood pressure and CVD risk factors across the ALT and GGT quartiles [5]. The study indicated GGT as a better marker for predicting the risks of CVD than other liver enzymes. In our study, serum GGT also showed the strongest association with CVD in both genders, which aligns with other studies. In a survey of British men, GGT showed a positive association among all‐cause mortality, ischemic heart disease‐related mortality and CVD risk factors [22]. In another longitudinal study, GGT was independently associated with mortality from CVD in Austrian adults [23]. A meta‐analysis of prospective studies showed that an increased 1 IU/L of GGT was associated with HR = 1.20 for chronic heart disease and HR = 1.54 for stroke [8]. Although previous studies demonstrated an independent association between liver enzymes and CVD risks, considerable heterogeneity was observed among the study's findings. Altogether, apart from hepatic diseases, growing evidence suggests that GGT is an effective marker for obesity, dyslipidemia, diabetes and hypertension [24, 25, 26, 27].

Although the mechanisms between elevated aminotransferases and increased risk of CDV remain unclear, NAFLD has been demonstrated as a possible explanation for the relationship between elevated aminotransferase and CVD mortality [28, 29] as it is involved with the hepatic manifestation of insulin resistance and metabolic syndrome [28, 30, 31]. However, our results show that increased AST and ALT levels are still related to the prevalence of CVD after adjusting for conventional risk factors. Several clinical bodies of evidence suggest that NAFLD is not only associated with liver‐related morbidity and mortality but also related to an increased risk of developing extrahepatic diseases such as CVD, T2DM, chronic kidney disease and extrahepatic cancers [32, 33, 34, 35]. Up to now, there is insufficient information on how NAFLD affects the relationship between aminotransferase levels and the risk of CVD events. It has been demonstrated that increased aminotransferase levels are associated with a higher risk of CVD through inflammation, endothelial dysfunction and reduced haemostasis [12, 32]. Some studies showed that aminotransferase concentrations, even within the normal range, are related to inflammatory and atherosclerosis markers [36, 37].

On the contrary, the underlying mechanisms for the link between elevated GGT and CVD risks are poorly understood. However, there are some potential explanations; first, GGT promotes low‐density lipoprotein (LDL) oxidation through a redox reaction, leading to the formation of atherosclerotic plaques, maturation and rupture [38]. In a study, active staining of GGT was found within atherosclerotic coronary plaques [39], suggesting an essential factor in addition to traditional CVD risk factors. Second, GGT is important in regulating glutathione, which is vital to human antioxidant defence [40, 41]. Third, GGT is considered a marker of oxidative stress and inflammation [40], essential CVD features. GTT‐mediated oxidative stress may influence plaque formation, erosion and rupture, increased platelet aggregation and thrombosis [38, 42]. In a longitudinal study, GGT was significantly associated with several inflammatory markers, including fibrinogen, hs‐CRP and F2‐isoprostanes [43].

Overall, there are inconsistent findings on the association between liver enzymes, especially aminotransferases (AST and ALT) and CVD risks. Our investigation adds to the evidence that in addition to GGT, increased levels of AST and ALT are also associated with the increased risk of CVD, and the association remains even after adjusting for potential confounders. This study has some limitations. First, the cross‐sectional data might affect the causal relationship between liver enzymes and CVD. Second, this study was limited to a small number of participants with MI; therefore, our results may not represent all patients with CVD in Bangladesh. Third, liver enzyme and other parameters measured at baseline were included in the analysis. Fourth, we did not have information about antihypertensive and antidiabetic medications specifically taken by patients with CVD that may affect the liver enzyme levels. Fifth, we could not collect information about dietary habits, family history of related diseases and medication history, which might affect liver enzyme levels. Lastly, this study is limited to the Bangladeshi population; therefore, our findings need to be reproduced in other cohorts in different clinical settings and ethnicities. Despite several limitations, the main strength of this study is that we evaluated the relationship between liver enzymes and CVD, including most of the relevant risk factors for CVD in the regression models. This study provides the first evidence of a link between serum liver enzymes and CVD in the Bangladeshi population, which may serve as a valuable reference for future research.

## 5 Conclusions

Our study shows a high prevalence of liver enzyme abnormalities in individuals with MI in the CVD group. The increased levels of AST, ALT and GGT were independently associated with a higher risk of CVD. Among these enzymes, GGT was found to be most strongly linked to CVD and therefore may be a better marker for assessing CVD risk. Overall, measuring the concentration of liver enzymes could be a valuable indicator to predict CVD in its early stages, as such patients are at an increased risk of liver dysfunction. Attention should be paid to monitor liver injury in patients with CVD. Further study is required to comprehend the underlying mechanisms connecting elevated liver enzymes to CVD, particularly MI.

## Author Contributions

**Akibul Hasan:** Data curation (equal); Investigation (equal); Methodology (equal); Writing – original draft (equal). **Ali Newaj:** Data curation (equal); Investigation (equal); Methodology (equal). **Aporajita Das Trisha:** Methodology (equal); Validation (equal). **Jaasia Momtahena Hafsa:** Investigation (equal); Methodology (equal). **Nayan Chandra Mohanto:** Investigation (equal); Methodology (equal). **Nurshad Ali:** Conceptualization (equal); Funding acquisition (equal); Investigation (equal); Methodology (equal); Supervision (equal); Validation (equal); Writing – review and editing (equal).

## Ethics Statement

The study protocol (ID 02/BMB/2019) was approved by the Ethics Review Committee of the BMB Department, School of Life Sciences, SUST. All study subjects provided written informed consent before study commencement.

## Conflicts of Interest

The authors declare no conflicts of interest.

## Data Availability

The data that support this study's findings are available from the corresponding author upon reasonable request.
